# Bilateral Tonsillitis With Peritonsillar Abscess

**DOI:** 10.7759/cureus.17546

**Published:** 2021-08-29

**Authors:** Kyra Alston, Rachel Sklar, Thor S Stead, Carlos O Lopez Ortiz, Latha Ganti

**Affiliations:** 1 Emergency Medicine, Trinity Preparatory School, Winter Park, USA; 2 Emergency Medicine, Brown University, Providence, USA; 3 Medicine, Warren Alpert Medical School, Brown University, Providence, USA; 4 Emergency Medicine, Ocala Regional Medical Center, Ocala, USA; 5 Emergency Medicine, University of Puerto Rico, Medical Sciences Campus, San Juan, PRI; 6 Emergency Medicine, Envision Physician Services, Plantation, USA; 7 Emergency Medicine, University of Central Florida College of Medicine, Orlando, USA; 8 Emergency Medicine, HCA Healthcare Graduate Medical Education Consortium Emergency Medicine Residency Program of Greater Orlando, Orlando, USA

**Keywords:** recurrent tonsillitis, peritonsillar abscess, amoxicilllin-clavulanate, pharyngitis, staphylococcus

## Abstract

The authors present a case of an adolescent female who presented to the emergency department with a second case of tonsillitis in the span of two weeks. The patient recovered after treatment with a broader-spectrum antibiotic and was discharged home. The authors highlight the importance of weighing the costs and benefits of tonsillectomy with the potential that additional antibiotics may be enough in cases of recurrent tonsillitis.

## Introduction

Tonsillitis is a common acute presentation, consisting of 1.3% of all outpatient visits in the emergency department (ED) [1]. Exposure is a key component in the spread of tonsillitis, and recurrent tonsillitis (RT) in particular [2]. Consequently, it is typically found in children and adolescents, and transmitted in places where children may expose one another to different illnesses, such as playgrounds and schools.

Tonsillitis is generally the result of an infection, presenting as viral or bacterial. Viral tonsillitis is more common, but not as severe as bacterial tonsillitis, with viral tonsillitis dissipating in a week and bacterial tonsillitis taking longer. The most common etiology of non-recurrent bacterial tonsillitis is Group A Streptococcus. However, the etiology of recurrent bacterial tonsillitis is most often *Staphylococcus aureus* (S. aureus). *S. aureus* is frequently found in patients with RT due to its antimicrobial resistance, which enables it to thrive in tonsil tissue [3]. Unlike viral infections, which are best left alone, bacterial tonsillitis is treated with antibiotics.

However, if the strain proves resistant or recurs, a tonsillectomy is advised. Tonsillectomies are effective in improving quality of life and health in adults with chronic tonsillitis and/or RT [4]. In this paper, the authors present a case of recurring bilateral bacterial tonsillitis: the pathology, effect on patients, and treatment modalities.

## Case presentation

**Figure 1:**
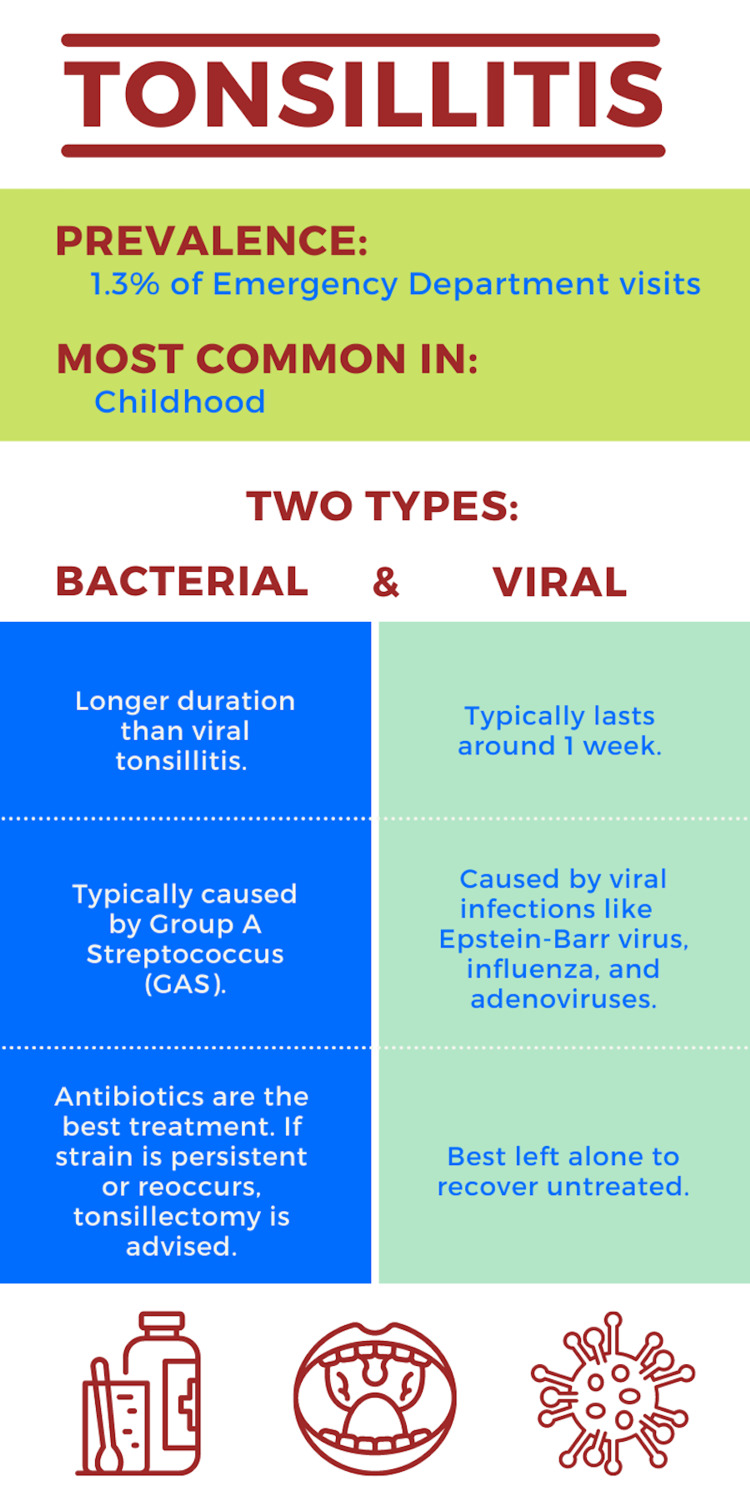
Infographic depicting features of bacterial and viral tonsillitis

An 18-year-old female with a history of RT presented with a two-day history of bilateral tonsillar swelling, with accompanying sore throat, difficulty swallowing, pharyngeal exudates, and a hot potato voice, features common to tonsillitis (Figure 1).

Surgical history included only a breast abscess drainage. Two weeks prior, she was found to have bilateral tonsillitis, successfully treated with a 10-day course of amoxicillin before arrival to the ED.

**Figure 2:**
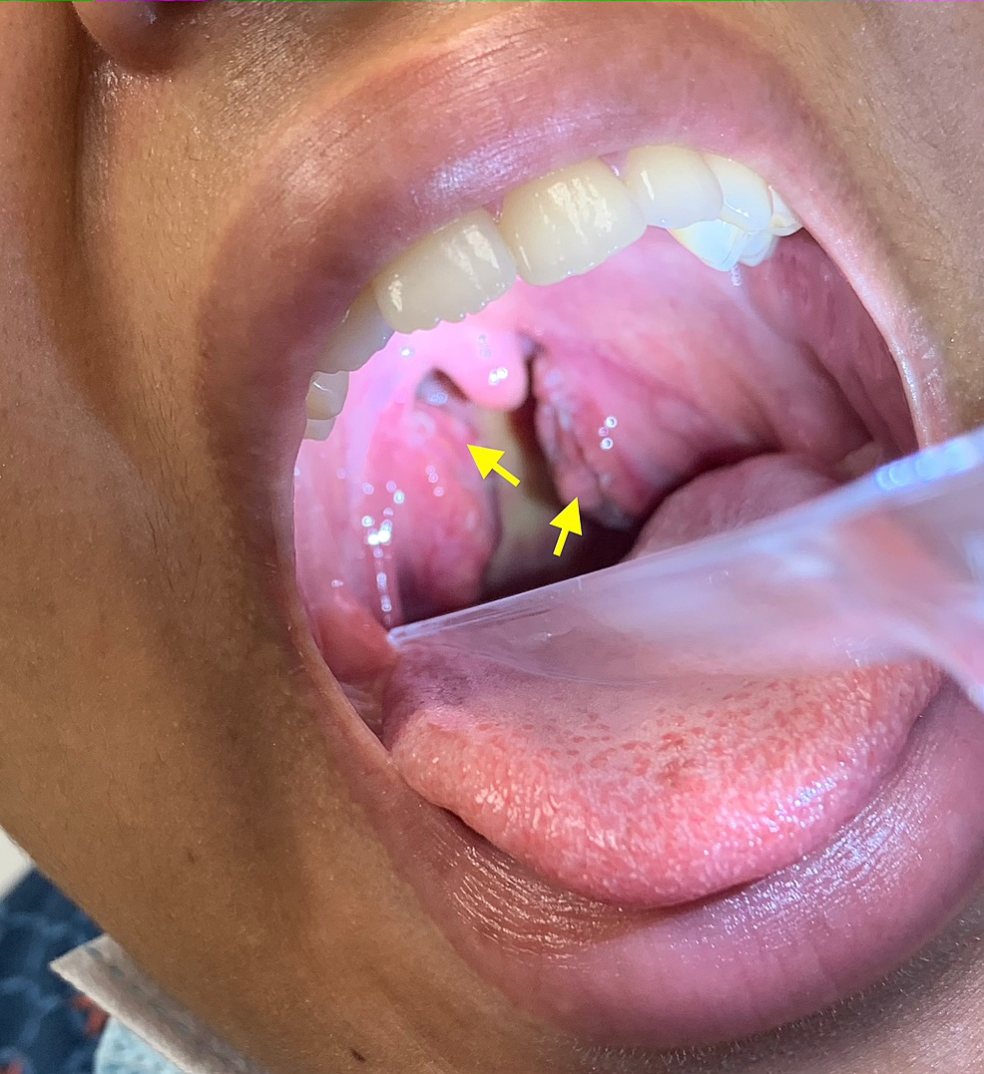
Clinical photograph of patient's oropharynx

On clinical examination, her vitals were slightly abnormal, with a fever of 100.7°F, a corresponding pulse of 104 beats per minute, and an elevated blood pressure at 135/70 mmHg. Neck examination revealed lymphadenopathy. Ears, nose, and throat examination revealed pharyngeal erythema, bilateral tonsillar erythema, bilateral tonsillar exudate, bilateral tonsillar swelling, and a left peritonsillar abscess (Figure 2).

**Figure 3:**
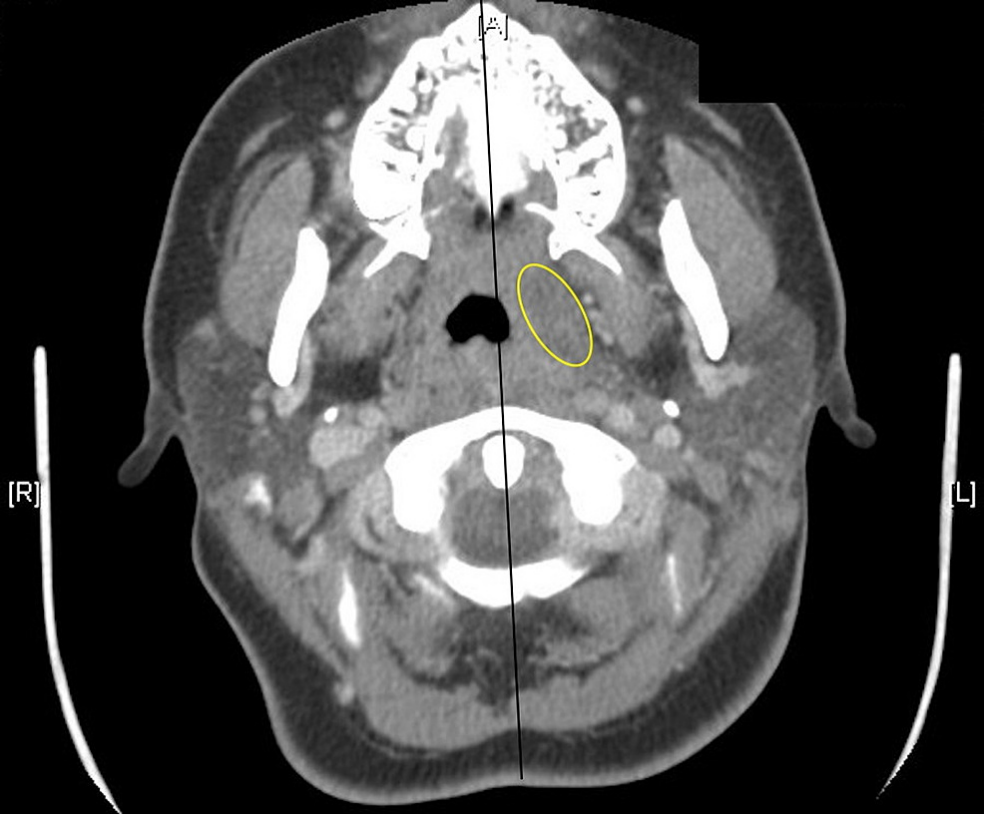
Axial-view computed tomography image demonstrating midline shift and tonsillar abscess on the left (yellow oval)

The patient had an elevated white blood cell count in response to the tonsillar infection (Table 1). Head and neck computed tomography scans revealed moderate heterogeneous prominence of the bilateral palatine tonsils with a small tonsillar abscess (15 mm hypodensity on the left) (Figure 3).

**Table 1:** Patient’s laboratory results

Chemistry	
Sodium (135-145 mmol/L)	137
Potassium (3.5-5.3 mmol/L)	3.9
Chloride (98-107 mmol/L)	99
Carbon Dioxide (21-32 mmol/L)	28
Blood Urea Nitrogen (7-18 mg/dL)	14
Creatinine (0.6-1.3 mg/dL)	0.9
Glucose (74-106 mg/dL)	90
Calcium (8.4-10.2 mg/dL)	9.5
Hematology	
White Blood Cell Count (4.1-9.3 k/mm^3^)	15.8 (H)
Red Blood Cell Count (3.28-5.50 M/mm^3^)	4.87
Hemoglobin (12.1-15.1 g/dL)	13.4
Hematocrit (35.5-46.9%)	40.6
Platelet Count (150-450 k/mm^3^)	323

**Figure 4:**
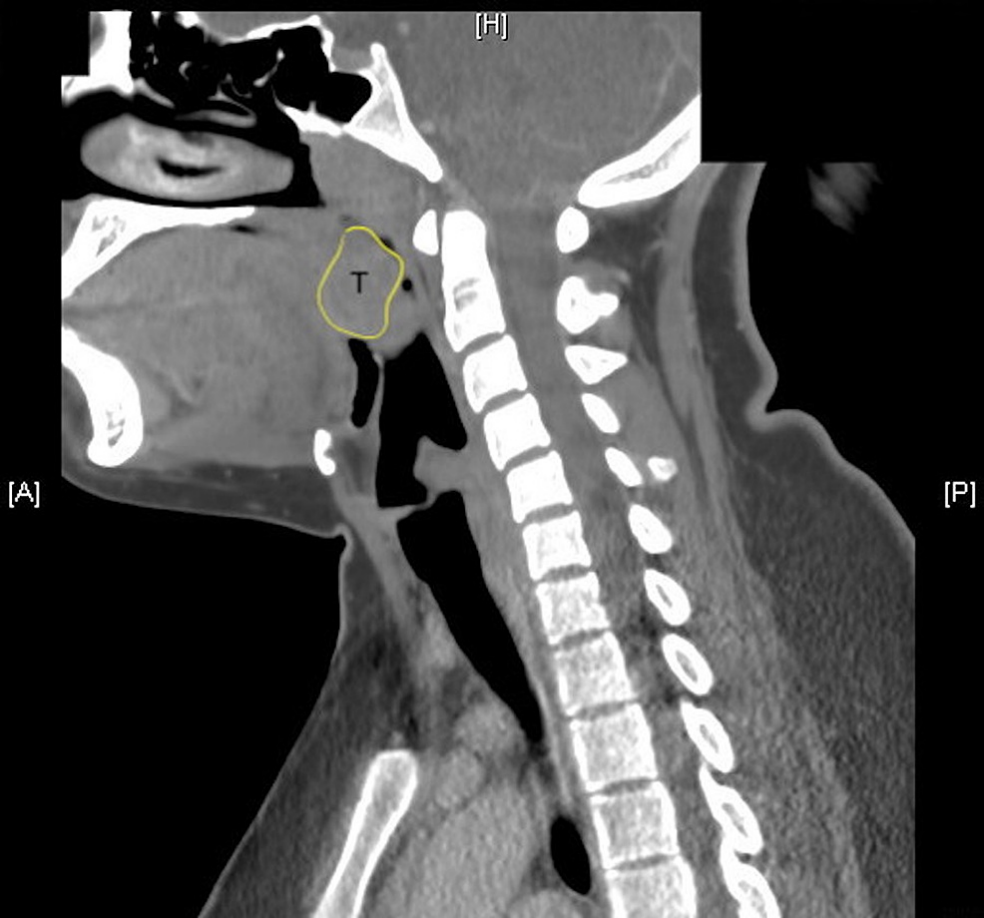
Saggital-view computed tomography image demonstrating tonsillar abscess (T)

Also noted is an esophageal diverticulum (3.5 cm) and moderate somewhat symmetric bilateral neck lymphadenopathy particular to the submandibular regions with tonsillar abscess (Figure 4).

The patient was admitted to the hospital, seen by otorhinolaryngology, and treated with amoxicillin-clavulanate. Her symptoms subsided over a few days and the patient was discharged home.

## Discussion

This patient presented with a second case of tonsillitis in two weeks, suggesting that the initial antibiotics either did not resolve the incident, or the patient was infected with tonsillitis again after the conclusion of the treatment plan. The patient was treated with amoxicillin during the first episode and amoxicillin-clavulanate on the second occurrence. Amoxicillin-clavulanate was selected to treat the second episode because it is a broader-spectrum antibiotic than amoxicillin. Clavulanate has superior anti-anaerobic, anti-Gram-negative, and anti-Gram-positive coverage; it successfully treated the episode. 

Though having two episodes of tonsillitis in such a short time frame is a cause for concern, it does not meet the definition for severe tonsillitis. According to current research, severe tonsillitis is defined as five or more separate presentations of tonsillitis in a year. The definition further includes episodes of tonsillitis that prevent the patient from functioning normally. Cases of tonsillitis caused by infectious mononucleosis are not included in the count for severe tonsillitis, as they are isolated incidents [5].

This patient missed many days of school due to her illness. This can cause a decreased quality of life in adolescents, with potential serious academic and social consequences. A United Kingdom study found that adult patients with RT report a low disease-specific quality of life, which is improved after tonsillectomy. In this population, they had an average of 27 episodes of tonsillitis prior to the tonsillectomy. On average, they waited seven years for the surgery [6]. In an otherwise healthy adolescent patient like this one, there would be significant disease-specific quality-of-life challenges and missed academic and professional opportunities if practitioners waited this long to complete a tonsillectomy. However, the costs and risks associated with completing an unnecessary tonsillectomy for this patient, who had only had two episodes at this point, outweighed the potential benefits.

In cases of tonsillitis where tonsillectomy is being considered, the risks and benefits of tonsillectomy must be weighed over other treatment options. Studies have long shown that while on waitlists for the tonsillectomies, some patients resolve on their own and no longer need the surgery by the time they are at the top of the list. Specifically, studies on RT in children have found that 19-27% of those on waitlists to receive tonsillectomy recover before they receive the surgery. The wait times were on average 10.6 months and longer than nine months, respectively [7-9]. These studies suggest that RT, even severe enough to warrant discussion of tonsillectomy, may resolve on its own given time.

This patient’s tonsillitis resolved on its own, consistent with the results of these studies. At this time, the patient’s tonsillitis is not yet defined as severe tonsillitis as it has only been diagnosed twice, though only time will tell if the patient’s tonsillitis reappears.

## Conclusions

This case highlights the prevalence of RT. It reminds practitioners of the importance of careful analysis when deciding whether to perform a tonsillectomy or give an additional dose of antibiotics when it presents. Factors to consider include number of episodes, potential to try a more broader-spectrum antibiotic, and the patient’s disease-specific quality of life. 
